# Place of mTOR inhibitors in management of BKV infection after kidney transplantation

**DOI:** 10.15171/jnp.2016.01

**Published:** 2015-12-20

**Authors:** Thomas Jouve, Lionel Rostaing, Paolo Malvezzi

**Affiliations:** ^1^Clinique Universitaire de Néphrologie, CHU Grenoble, France; ^2^Université Grenoble Alpes, Grenoble, France; ^3^Department of Nephrology and Organ Transplantation, CHU Rangueil, Université Paul Sabatier, Toulouse, France; ^4^INSERM U563, IFR–BMT, CHU Purpan, Toulouse, France

**Keywords:** Kidney transplantation, BK virus infection, BKV-associated nephropathy, Sirolimus, Everolimus, Immunosuppression

## Abstract

*Context:* BK virus (BKV) viremia and BKV-associated nephropathy (BKVAN) have become a serious nuisance to kidney transplant (KT) patients since the mid-nineties, when the incidence of this disease has increased significantly.

*Evidence Acquisition:* Directory of open access journals (DOAJ), EMBASE, Google Scholar, PubMed, EBSCO, and Web of Science have been searched.

*Results:* Many hypothesis have been made as to why this phenomenon has developed; it is of general opinion that a more potent immunosuppression is at the core of the problem. The use of the association of tacrolimus (TAC) with mycophenolic acid (MPA) has gained momentum in the same years as the increase in BKV viremia incidence making it seem to be the most likely culprit. m-TOR inhibitors (m-TORIs) have been shown to have antiviral properties in vitro and this fact has encouraged different transplant teams to use these agents when confronted with BKV infection (viremia or nephropathy). However, the results are mitigated. There had been conflicting results for example when converting from TAC-to sirolimus-based immunosuppression in the setting of established BKVAN.

*Conclusions:* In order to prevent BKV infection we have to minimize to some extent immunosuppression, but it is not always possible, e.g. in high immunological risk patients. Conversely, we could use m-TORIs associated with low-dose calcineurin inhibitors (CNIs). This could be actually the key to a safe immunosuppression regimen both from the immunological stand point and from the viral one.

**Implication for health policy/practice/research/medical education:**

In order to prevent BK virus (BKV) infection we have to minimize to some extent immunosuppression, but it is not always possible, e.g. in high immunological risk patients. Conversely, we could use m-TOR inhibitors (m-TORIs) associated with low-dose calcineurin inhibitors (CNIs). This could be actually the key to a safe immunosuppression regimen both from the immunological stand point and from the viral one.

## 1. Context

BK virus (BKV) belongs to the family of polyomaviridae, a group of small double-stranded DNA viruses (1). Unapparent spread of infection occurs early in childhood and seroprevalence among the general population is high at ≈80% (2,3). The virus has a specific tropism for the urogenital epithelium that represents a site of viral latency. BKV-associated pathologies mainly occur in immunocompromised patients.

## 2. Evidence Acquisition

For this review, we used a variety of sources by searching through PubMed/Medline, Scopus, EMBASE, EBSCO and directory of open access journals (DOAJ). The search was conducted, using combinations of the following key words and or their equivalents; kidney transplantation; BKV infection; BKV-associated nephropathy; sirolimus; everolimus and immunosuppression.

## 3. Results

In the setting of organ transplant patients, BKV infection occurs mainly in kidney transplant (KT) patients; in that group of patients the prevalence of viruria, viremia and BKV-associated nephropathy (BKVAN) is as high as 30%, 13%, and 8% respectively (4). It is still not clear whether reactivation of latent BKV is host- or donor-derived. BKV infection will result in tubulointerstitial nephritis and in some cases in ureteral stenosis with a high risk of subsequent allograft loss in 15%-50% of cases (5,6).

Risk factors for having a treatment for BKV infection include the use of antithymocyte globulins as part of the induction scheme, receiving tacrolimus (TAC)-based immunosuppression instead of cyclosporine A (CsA)-based immunosuppression, having MMF-containing regimen instead of no anti-metabolite-containing regimen, and steroid therapy; conversely sirolimus (SRL)-based immunosuppression as opposed to CsA-based immunosuppression had a protective effect (7). Other risk factors include recipient and donor ages, race (white), gender (male), HLA-mismatches, previous biopsy-proven acute rejection and ureteral stent placement (8,9). To date, there is no effective antiviral therapy when BKVAN is present. Management of patients affected by BKVAN mainly relies on reducing the total immunosuppression (10). Cidofovir treatment has been attempted; however its nephrotoxicity limits its clinical use (11). Another strategy would be to modify immunosuppression when BKV infection occurs by eliminating mycophenolic acid (MPA) and replacing it by m-TOR inhibitors (mTORIs) and decreasing calcineurin inhibitor therapy or converting from TAC to CsA. Hence mTORIs may have anti-BKV properties.

BKV-specific T-cell responses, and particularly BKV-specific interferon (IFN)-γ−producing T-cells are markers of antiviral immune protection (12,13). Egli et al reported that BKV-specific T-cell response in vivo correlated significantly with TAC trough levels, but not with mycophenolate levels, prednisone dosing or the overall immunosuppression (14). Indeed, Comoli et al reported that reducing immunosuppression was associated with an increase of the BKV-specific cellular immune responses (15). Egli et al observed that patients with TAC levels below 6 ng/ml had significantly higher BKV large T-antigen specific activity compared to patients with TAC levels above 6 ng/ml (14). The relevance of this observation is underlined in a study where high BKV large –antigen specific cellular immune responses were associated with more than 2 log_10_ BKV decreasing and clearing plasma viral loads (12,16). Egli et al also demonstrated that at clinically relevant concentrations, SRL, MPA or leflunomide did not show a significant inhibition of BKV-specific T-cell activation and IFN-γ release (14). Corresponding results were also obtained by means of BKV-specific interleukin-2 and tumor necrosis factor-α secretion. However, the addition of SRL during BKV-antigen-specific expansion and re-challenge with BKV antigen revealed that antigen-specific expansion and not the overall T-cell activation was affected by mTORIs (14). In addition, SRL may be associated with lower incidence rate of BKVAN, even when combined with low-dose CNIs (17,18).

### 3.1. Factors contributing to infection with the BK virus

Many factors contribute to infection with the BKV, with one of the most important being the type of immunosuppression. Recently, Hirsch et al reported on more than 600 de novo KT patients who were randomized at pre-transplant to received either TAC-based or CsA-based immunosuppression (1:1 ) as part as the Diabetes Incidence after Renal Transplantation: Cyclosporine C2 monitoring versus Tacrolimus (DIRECT) study in which BKV viremia and viruria were prospectively centrally monitored for up to 12 months post-transplantation (19). Kaplan–Meier statistics showed that the incidence of new-onset BKV viruria and viremia at month 12 was up to 39.5% (95% CI: 35.4%–43.5%) and 23.9% (95% CI: 20.4%–27.3%), respectively. The highest rates of viruria and viremia were observed at month 6 (25.4% and 13.7%, respectively). Biopsy-proven acute rejection episodes were also significantly more frequent in patients with BKV viremia at month 6 (13% versus 6.1%; *P *= 0.03), and at month 12 the estimated glomerular-filtration (eGFR) rate was significantly lower in viremic patients (median e-GFR 60.4 ml/min) compared to non-viremic patients (median e-GFR 65.7 ml/min; *P *= 0.032). With regards to the type of calcineurin inhibitors (CNIs), patients receiving CsA had a lower rate of viremia compared to those that received TAC at both month 6 (10.6% versus 16.3%; *P *= 0.048) and month 12 (4.8% vs. 12.1% ; *P *= 0.004). In addition, high-level viremia of >4 log_10_ copies/ml was lower in patients receiving CsA (2.2%) than in those that received TAC (9.4%; *P *< 0.0001) at month 12. Moreover, median plasma BKV loads were 15-fold (1.2 log_10_ copies/ml) lower in those that received CsA rather than TAC (*P *= 0.028). This large prospective randomized controlled trial showed, for the first time, that BKV infection was significantly more prevalent within the first year post-transplant in TAC-treated patients compared to patients that received CsA (19).

When BKV infection occurs there is no definite strategy. Schaub et al reported on the outcomes of 38 KT patients with a BKV infection and whose immunosuppression was decreased after diagnosis (20). Of these, 13 had definitive BKVAN, 17 had presumptive BKVAN defined by plasma BKV loads of ≥4 log_10_ copies/ml, and 8 had low BKV viremia. In all patients with sustained BKV viremia, immunosuppression was reduced as follows: Step 1: TAC trough levels were reduced from 8–10 ng/ml (as intended in the protocol) to 6–8 ng/ml. Step 2 was implemented if BKV viremia did not decrease, for example TAC trough levels were reduced with a further reduction from 6–8 to 4–6 ng/ml. Finally, if BKV viremia was still not decreased, then step 3 was implemented, i.e., MPA was reduced by 50%. In 45% of patients TAC trough levels were reduced by one step, in 34% TAC trough levels had to be reduced by two steps; in the other cases either there was no additional reduction in immunosuppression (n* *= 3) or other intervention were made (n* *= 5; mainly elimination of MMF replaced by SRL). During follow-up, 8.6% of patients presented with biopsy proven acute rejection. Clearance of BKV viremia was observed in 92% of patients, with frequencies of clearance not different across the three groups. Interestingly the median time from first BKV viremia to clearance of BKV viremia was 8.8 months in the definitive BKAN, 4.6 months in the presumptive BKAN, and 2.9 months in the low BKV viremia group (*P *= 0.001). Reduction of immunosuppression to achieve clearance of BKV viremia was most extensive in patients with definitive BKVAN. At diagnosis as well as at last follow up serum creatinine was not statistically different across the three groups. Finally after a median follow-up time of 34 (range 18-60) months there was no allograft loss (20). The conclusion from that prospective study are twofold: (*i*) BKV monitoring at blood level in de novo KT patients is worth its cost, and (*ii*) when BKV infection occurs reducing immunosuppression will result in clearing the virus even though it takes months. This was achieved without impairment of renal function. However, long-term data in these patients are necessary particularly with regards to development of de novo DSA.

### 3.2. BKV and mTOR inhibitors

### 3.2.1 Background

**Figure 1:**
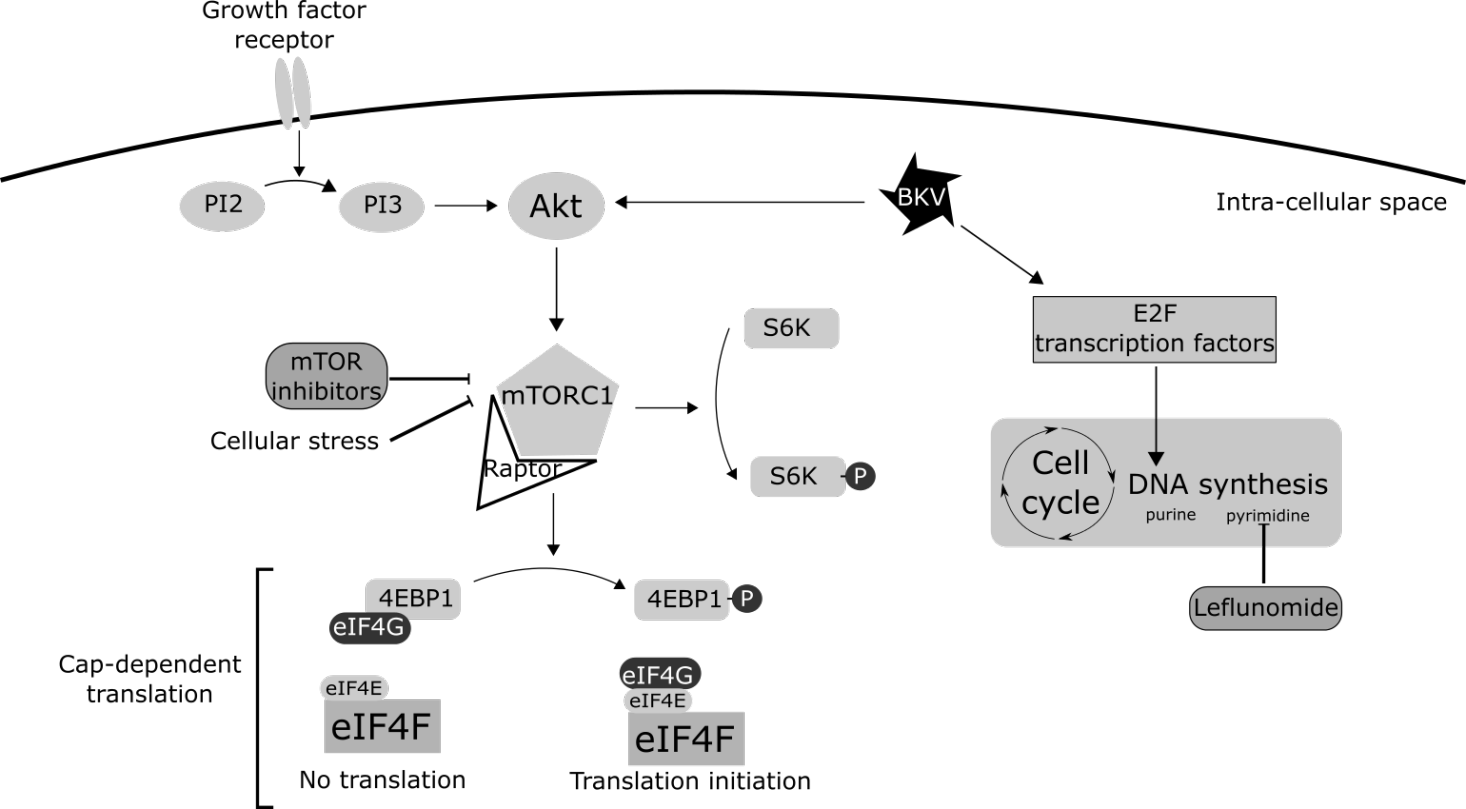
BK virus interactions with the cell machinery. A simplified version of the Akt-mTOR-eIF4 pathway is represented. mTOR inhibitors- and leflunomide-targeted points are outlined.

As a virus, BKV relies on the host’s cellular machinery to replicate. Upon entry into a cell, BKV induces cellular stress. This stress, for example, caused by the accumulation of viral proteins or a deficiency of amino acids, down-regulates DNA replication and can then induce apoptosis or necrosis. In order to replicate efficiently, BKV must balance this induced cellular stress. Viruses use several mechanisms to bypass these stress signals and slow or halt translation or DNA replication. These mechanisms involve two main pathways: the eukaryotic initiation factor 4 (eIF4) complex (21) and the E2F family of transcription factors (22) (Figure 1).

The eIF4 complex is at the heart of cap-dependent translation, enables replication of double-stranded DNA viruses, and is a target for TOR inhibitors. Briefly, eIF4 function is regulated by the phosphatidylinositol triphosphate kinase (PI3K)–Akt–mTOR pathway. In this pathway, PI3K increases the cellular content of PI3, which activates Akt; Akt itself then activates mTOR. The eIF4 complex requires an interaction between the different subunits, eIF4E and eIF4G, in order for translation to occur. This interaction is inhibited by the eIF4E-binding protein (4E-BP1), which is a target in the mTORC1 complex. mTORC1 phosphorylates 4E-BP1, thus preventing 4E-BP1 from binding to eIF4G and thereby maintains activity of the eIF4 complex and allows translation to occur (23).

The viral mechanisms that maintain cap-dependent translation act on Akt at the beginning of the mTOR pathway. This allows mTOR inhibitors to restore downregulation of the translation that occurs under cellular stress, thereby slowing viral replication.

Furthermore, in vitro studies have focused on leflunomide (a pyrazinamide-synthesis inhibitor) as a treatment for BKV infections. The combination of an mTOR inhibitor and leflunomide effectively limits viral DNA replication and thus limits synthesis of viral proteins. This combination is stronger than for any mTOR inhibitor alone, showing that BKV uses different pathways and not only the PI3K–Akt–mTOR pathway for transcription enhancement (24).

In vitro studies on BKV’s response to different immunosuppressive drugs, including CNIs and mTOR inhibitors, show that SRL does not inhibit BKV-specific T-cell activation, in contrast to CNIs. However, SRL inhibits the mTOR dependent proliferation of these T-cells (14).

Finally, mTOR inhibitors regulate the differentiation of memory CD8 T-cells (25,26), improving the immune reaction against BKV after infection.

### 3.2.2. Clinical evidence

During the period 2003–2006, Dharnidharka et al reported on the relative risk of BKV infection treatment within the first 24 months after kidney transplantation, using data from the OPTN registry (17). They noted a significantly lower cumulative incidence of BKV treatment in primary KT patients receiving mTOR inhibitors at hospital discharge (n = 5380) compared to patients receiving other regimens without an mTOR inhibitor (n = 42 912; 1.74% vs. 3.67%; *P *< 0.001), with this difference sustained in multivariate analysis (hazard ratio [HR]: 0.69 [0.59–0.89]).

Conversely, in a single-center retrospective study, Gralla and Wieseman reported on BKV infection (diagnosed by a biopsy and viremia) in 518 consecutive de novo KT patients who received a transplant between 2000 and 2006 and whose immunosuppression relied either on TAC + SRL or TAC + mycophenolate mofetil. A BKV event occurred in 3.8%–7.4% of patients receiving mycophenolate mofetil and in 5.9%–6.9% of patients receiving SRL (*P *= N.S) (27). Analysis of data collected between 2004 and 2006 from the Scientific Renal Transplant Registry on 42 838 de novo KT patients showed that the outcomes of treated-BKV infection within the first 12 months post-transplant were similar in patients treated with SRL (adjusted odds ratio [AOR]: 0.7; 95% CI: 0.47–1.03) compared with those that received CsA or TAC (AOR: 1.35; 95% CI: 1.04–1.74) (28).

### 3.3.3. BKV infection and sirolimus

Wali et al reported on a rescue therapy using SRLbased immunosuppression given to three patients with BKVAN (29). The authors initially performed protocol kidney-allograft biopsies on 25 patients with progressive allograft dysfunction and, unexpectedly, three showed BKVAN at 16–36 months posttransplant. All three patients were receiving a maintenance therapy of steroids/mycophenolic acid/ TAC. TAC and MPA were withdrawn in all three, and replaced by SRL to aim at trough levels of ~10–12 ng/ml. At the time of conversion, median BKV viremia was 60 303 copies/mL (12 481–326 117). Viremia decreased by more than 50% during the first 2 months after SRL therapy and was almost undetectable at 19 months post-conversion; renal function also concomitantly improved, i.e., median e-GFR increased from 52 to 67 ml/min (29).

Jacobi et al reported on the potential role of m-TORIs to treat BKV infections in KT recipients (30). This retrospective cohort study involved 352 de novo KT patients who had been screened post-transplantation for BKV viremia at months 3, 6, 9, and 12. In addition, the patients had protocol biopsies at post-op months 3 and 12. Immunosuppression was based on CNIs, MPA, plus steroids. In cases where there were low levels of BKV viremia and no features of BKVAN on the allograft biopsy, baseline immunosuppression was reduced (CNIs by 30% and MPA by 50%). In the other settings, i.e. further rise in BKV viremia despite decrease in immunosuppression or biopsy-proven BKVAN, patients were converted from TAC to CsA (Co level: 60–80 ng/ml) plus mTOR inhibitors (trough level: 5–8 ng/ml). During the first year post-transplant, BKV viremia was detected in 13.6% of patients; in 22 of these 48 patients kidney biopsies showed BKVAN. The mean onset of BKV viremia was at around 180 days post-transplant: four patients lost their graft because of BKVAN. In patients with BKV viremia without BKVAN, renal function remained stable within the first year after diagnosis, irrespective of the treatment for BKV infection. In contrast, renal function in patients with BKVAN deteriorated over time; however, in the longer term, renal function was better in those where TAC was replaced by CsA with the addition of mTOR inhibitors compared to those where CNIs and MPA were reduced, i.e., without adding mTOR inhibitors (30).

Recently, Tohme et al reported on a single-center 3-year study that evaluated BKV infection in 180 KT patients whose immunosuppression relied on either TAC or SRL (31). The study was conducted between 2008 and 2011. One group of patients received TAC plus mycophenolate mofetil without steroids and was not evaluated for BKV infection; the second group received TAC/mycophenolate mofetil/steroids (n = 78). The third group (the SRL group) received TAC/mycophenolate mofetil/steroids for up to 3 months post-transplant. After this time, TAC was stopped and replaced by SRL (to target trough levels at >5 ng/ml; n = 47). Patients with a high immunological risk received anti-thymocyte globulins as the induction therapy whereas the others received basiliximab as the induction therapy. In that study, BKV viremia was prospectively searched for at 3, 6, 12, and 24 months post-transplant using PCR. Clinically significant BKV viremia was defined as having a value of >10 000 copies/ml. BKV viral load was then repeated every 1-2 months, and was managed as follows: the dose of mycophenolate mofetil was halved as the first step; the next step was to add leflunomide and/or discontinuing mycophenolate mofetil when there was no response to the reduction in immunosuppression. The incidences of any detectable BKV viremia or of clinically significant BKV viremia were 35.9% and 17.9% in those that received TAC and 19.1% (*P *= 0.04) and 4.3% (*P *= 0.02) in the SRL group. Indeed, in the SRL group, all patients with clinically significant BKV viremia already had BKV viremia prior to conversion from TAC to SRL. In multivariate analysis, the only two independent factors associated with BKV viremia were male gender (HR [hazard ratio]: 2.87 [1.10–7.45]; *P *= 0.03) and SRL use (HR: 0.33 [0.12–0.96]; *P *= 0.04). Only two patients developed BKVAN: both had received TAC and both lost their allograft. Of note, after month 3 in the SRL group, only one patient, who was receiving SRL, developed BKV viremia, whereas patients on TAC continued to develop BKV viremia at different time points. Finally, mean peak viral load was higher in patients that received TAC (6.1 log_10_ copies/ml) than in those receiving SRL (3.8 log_10_ copies/mL); however, this difference was not statistically significant. Patient- and graft-survival rates, as well as the number of acute-rejection episodes, were similar across the two groups, whereas GFR was significantly higher at all times points in those that received SRL (31).

### 3.3.4. BKV infection and everolimus

Tedesco Silva et al reported, in 2010, the 1-year results of patients receiving everolimus plus reduced-exposure to cyclosporine versus MPA plus standard exposure to cyclosporine A in a total of 833 de novo KT patients (32). In this study, BKV infections were reported by the investigators as adverse events. They observed a higher incidence of BKV viruria and BKV viremia in the MPA patients (3.3% and 1.8%) than in the everolimus 1.5 mg patients (0.7% and 1.1%). Furthermore, there were three cases of BKVAN: one in the everolimus 1.5 mg group and two in the MPA group.

Moscarelli et al demonstrated that everolimus-based immunosuppression, compared to mycophenolic-acid-based immunosuppression, was associated with significantly less BKV viremia in KT recipients (33). They performed a single-center observational study of 296 de novo KT recipients between 2005 and 2010; of these, 66 received everolimus (trough levels of >3 ng/ml) plus low doses of CsA, or MPA plus full doses of CsA (n = 238). In addition, patients in both groups received basiliximab as an induction therapy plus a maintenance steroid therapy. BKV viremia was prospectively assessed by real-time PCR on a weekly basis between months 1 and 4, and then monthly. If BKV viremia was detected in the MPA group, no change in immunosuppression was attempted. A renal-allograft biopsy was performed if BKV viremia and allograft dysfunction were detected. The authors found that the frequency of BKV viremia was significantly higher in the MPA group than in the everolimus group (52.77% versus 59%; *P *= 0.01). The adjusted HR showed that the MPA group had a significantly higher risk of BKV viremia (HR: 1.71; 95% CI: 1.08–2.69; *P *= 0.02). Although, the meantime until the onset of BKV viremia was similar across the two groups (MPA: 3.8 ± 1 months; everolimus: 4.1 ± 1.5 months), the mean viral load at diagnosis was significantly higher in the MPA group (12.5 ± 6.1 × 10^4^ copies/ml) compared to the group that received everolimus (2.5 1.8 × 10^4^ copies/ml; *P *= 0.01). In addition, the time to clear BKV load was significantly shorter in the everolimus group (1 ± 0.2 month) than in the MPA group (10.7 ± 8 months; *P* < 0.01). Moreover, in the group that received everolimus, no patient developed BKVAN, whereas this occurred in 15 cases of the entire MPA group. Although the rates of acute rejection were similar across the two groups before the onset of BKV viremia, this was not the case after this time: hence none of BKV viremia-positive everolimus group presented with an acute rejection after the onset of BKV viremia, whereas this occurred in 9 patients in the MPA group after discontinuation of MPA. Allograft loss after diagnosing BKV viremia never occurred in the group that received everolimus, whereas this occurred in nine MPA group patients: five because of BKVAN and six because of an acute rejection (33).

## 4. Conclusions

BKV replication assessment at regular intervals during at least the first year post-transplantation is mandatory, particularly in those patients whose immunosuppression is based on TAC, and especially when targeted trough levels are above 6 ng/ml. When BVK replication occurs, a kidney allograft biopsy is required in order to check for the presence of BKVAN. When we face BKV infection we can either drastically reduce immunosuppression with regards to CNI whole blood concentrations and MPA dosage or we can modify immunosuppression by replacing MPA by an mTORi and minimizing CNI. Because the association of SRL to CNI is nephrotoxic, we would prefer the association of low dose-everolimus plus low-CNI. Indeed, in vitro data showed that mTORIs inhibit to some extent BKV replication. However, there is a need for randomized control trials with the aim to reduce post-KT BKV reactivation with one immunosuppressive regimen containing low everolimus/low CNI, as compared to the standard of care that is based on TAC plus MPA.

## Authors’ contribution

All authors wrote the manuscript equally.

## Conflicts of interest

The authors declared no competing interests.

## Funding/Support

None.
